# Supplementary material for: Cultivation of Melilotus officinalis as a source of bioactive compounds in association with soil recovery practices
Source: Front Plant Sci. 2023 Sep 12;14:1218594. doi: 10.3389/fpls.2023.1218594 (PMC10523325; doi:10.3389/fpls.2023.1218594)
Supplement: Supplementary file 2 [file DataSheet_1.docx]

Supplementary Material

Cultivation of *Melilotus officinalis* as a source of bioactive compounds in association with soil recovery practices

Isabel Nogues, Laura Passatore, María Ángeles Bustamante, Emanuele Pallozzi, João Luz, Francisco Traquete, António E.N. Ferreira, Marta Sousa Silva^*^, Carlos Cordeiro

*** Correspondence:** Marta Sousa Silva: mfsilva@fc.ul.pt

# Supplementary Table

**Supplementary Table 1.** Detected metabolites in *M. officinalis* plants grown on Control, Inorganic, C-Low and C-High soils, analysed by FT-ICR-MS in positive (ESI^+^) ion analysis mode (aligned table). Experimental *m/z* is the *m/z* value detected by FT-ICR-MS, considering H^+^, Na^+^ and K^+^ as possible adducts; the neutral mass (Da) is the corrected mass of the compound without adduct; Compound Name (First annotation) is the first name that appears in the database for that *m/z* value; Compound Formula (Smart Formula) is the formula of the compound calculated by Bruker’s Metaboscape Smartformula tool; for each database, HMDB (Human Metabolome Database), PlantCyc, and LOTUS, is shown the compound ID in the database, the name, formula and the number of possible identifications (match count) in that database. The intensities of the compounds in each replica of the four groups is also shown.
